# Chloridobis(1,10-phenanthroline-κ^2^ *N*,*N*′)copper(II) tetra­kis­(nitrato-κ^2^ *O*,*O*′)(1,10-phenanthroline-κ^2^ *N*,*N*′)terbate(III)

**DOI:** 10.1107/S160053681101837X

**Published:** 2011-05-25

**Authors:** Guo-Ran Gao, Hong-Xue Man, Zhi-Chang Xiao, Yu-Heng Deng

**Affiliations:** a Department of Chemistry, Capital Normal University, Beijing 100048, People’s Republic of China

## Abstract

The title complex salt, [CuCl(C_12_H_8_N_2_)_2_][Tb(NO_3_)_4_(C_12_H_8_N_2_)], consists of discrete [CuCl(phen))_2_]^+^ cations and [Tb(NO_3_)_4_(phen)]^−^ anions (phen is 1,10-phenanthroline). The [CuCl(phen))_2_]^+^ cation contains a five-coordinate Cu^2+^ ion, ligated by two bidentate phen ligands and one Cl^−^ ion, exhibiting a distorted CuN_4_Cl trigonal–bipyramidal geometry. In the [Tb(NO_3_)_4_(phen)]^−^ anion, the Tb^3+^ ion is coordinated by one chelating phen ligand and four chelating nitrates, forming a distorted TbN_2_O_8_ bicapped dodeca­hedral configuration. The anions and cations are assembled into a three-dimensional network by weak C—H⋯Cl and C—H⋯O hydrogen bonds. There is also a significant π–π stacking inter­action, with a centroid–centroid distance of 3.635 (2) Å.

## Related literature

For studies on mixed-metal ionic adducts with [CuCl(phen)_2_]^+^, see: Beznischenko *et al.* (2009 ▶); Draper *et al.* (2004 ▶); Yang *et al.* (2004 ▶). For related structures, see: Frechette *et al.* (1992 ▶); Kepert *et al.* (1996 ▶); Niu *et al.* (1997 ▶); Wei *et al.* (2002 ▶).
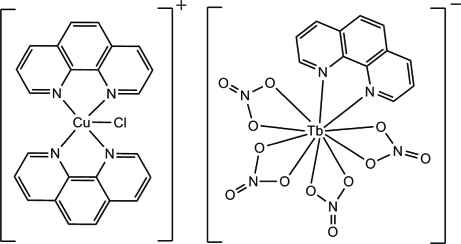


## Experimental

### 

#### Crystal data

[CuCl(C_12_H_8_N_2_)_2_][Tb(NO_3_)_4_(C_12_H_8_N_2_)]
*M* *_r_* = 1046.56
Triclinic, 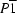

*a* = 10.1556 (1) Å
*b* = 13.4799 (2) Å
*c* = 14.7710 (2) Å
α = 81.103 (1)°
β = 75.631 (1)°
γ = 84.465 (1)°
*V* = 1931.71 (4) Å^3^
*Z* = 2
Mo *K*α radiation
μ = 2.51 mm^−1^
*T* = 296 K
0.21 × 0.20 × 0.12 mm

#### Data collection

Brucker SMART APEX CCD diffractometer
Absorption correction: multi-scan (*SADABS*; Bruker, 2007 ▶) *T*_min_ = 0.620, *T*_max_ = 0.753
25145 measured reflections
8895 independent reflections
6625 reflections with *I* > 2σ(*I*)
*R*_int_ = 0.040

#### Refinement

*R*[*F* ^2^ > 2σ(*F* ^2^)] = 0.035
*wR*(*F* ^2^) = 0.078
*S* = 1.03
8895 reflections
550 parameters
H-atom parameters constrained
Δρ_max_ = 0.44 e Å^−3^
Δρ_min_ = −0.75 e Å^−3^

### 

Data collection: *APEX2* (Bruker, 2007 ▶); cell refinement: *APEX2* and *SAINT* (Bruker, 2007 ▶); data reduction: *SAINT*; program(s) used to solve structure: *SHELXS97* (Sheldrick, 2008 ▶); program(s) used to refine structure: *SHELXL97* (Sheldrick, 2008 ▶); molecular graphics: *Mercury* (Macrae *et al.*, 2006 ▶); software used to prepare material for publication: *SHELXTL* (Sheldrick, 2008 ▶).

## Supplementary Material

Crystal structure: contains datablocks global, I. DOI: 10.1107/S160053681101837X/fj2415sup1.cif

Structure factors: contains datablocks I. DOI: 10.1107/S160053681101837X/fj2415Isup2.hkl

Additional supplementary materials:  crystallographic information; 3D view; checkCIF report

## Figures and Tables

**Table 1:** Hydrogen-bond geometry (Å, °)

*D*—H⋯*A*	*D*—H	H⋯*A*	*D*⋯*A*	*D*—H⋯*A*
C5—H5⋯Cl1^i^	0.93	2.64	3.551 (5)	167
C33—H33⋯O1^ii^	0.93	2.62	3.287 (5)	130
C2—H2⋯O2	0.93	2.47	3.302 (5)	149
C14—H14⋯O3^iii^	0.93	2.67	3.350 (5)	131
C26—H26⋯O4^iii^	0.93	2.57	3.468 (5)	162
C6—H6⋯O5^iv^	0.93	2.53	3.261 (5)	136
C9—H9⋯O6^v^	0.93	2.66	3.228 (6)	120
C22—H22⋯O9^v^	0.93	2.67	3.342 (6)	130
C1—H1⋯O10	0.93	2.58	3.377 (5)	144
C29—H29⋯O11^vi^	0.93	2.69	3.314 (4)	126

Symmetry codes: (i) 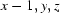; (ii) 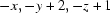; (iii) 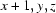; (iv) 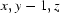; (v) 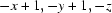; (vi) 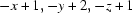.

**Fig. 1.**
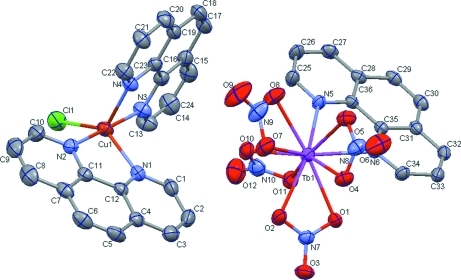
ORTEP drawing of title compound with the atom-numbering scheme, showing 30% probability displacement ellipsoids. (H atoms are omitted for clarity)

**Fig. 2.**
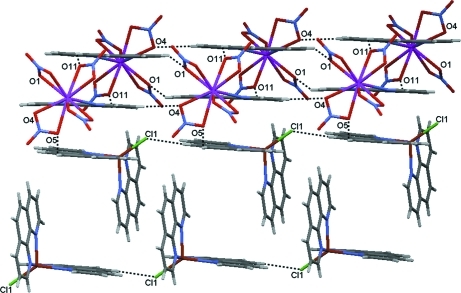
The cation and anion infinite chains along a axis formed by the hydrogen bonds and π–π stacking interactions.

**Fig. 3.**
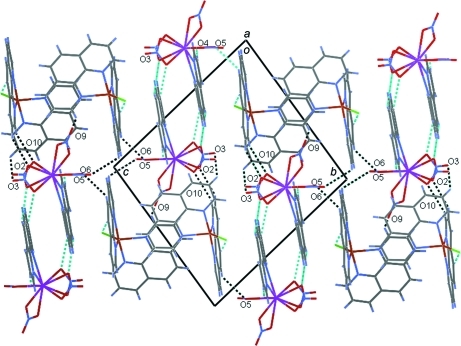
The packing diagram viewed along the a axis, showing the weak C—H···O hydrogen bonds between the cation and anion chains. (Shown as the black dashed lines).

## supplementary crystallographic information

### Comment

The title compound [CuCl(phen)_2_][Tb(NO_3_)_4_(phen)] is an unexpected product isolated from the reaction system of Tb(NO_3_)_3_.6H_2_O, Cu(ClO_4_)_2_.6H_2_O, 2-(Carboxymethylthio)benzoic acid and 1,10-phenanthroline in methanol solution.

There are few of mixed-metal ionic adducts similar to the title compound containing [CuCl(phen))_2_]^+^ ion reported now (Beznischenko, *et al.*, 2009; Draper, *et al.*, 2004; Yang, *et al.*, 2004]. Herein, we report a new ion adduct of Cu(II) complexcation and Tb(III) complexanion, [CuCl(phen)_2_][Tb(NO_3_)_4_(phen)]. The center Cu^2+^ ion of [CuCl(phen))_2_]^+^ is coordinated by two bidentate phen ligands and one Cl^-^ ion, exhibiting a distorted five-coordinated CuN_4_Cl trigonalbipyramidal geometry. However, the [Tb(NO_3_)_4_(phen)]^-^ anion is a distinct structure which is unique to the other lanthanide complexes with phen and NO^3-^. The center Tb^3+^ ion is coordinated by one phen and four nitrates, forming a conventional ten-coordinated dicapped dodecahedral configuration. But in the previous literatures, all the ten-coordinated mononuclear lanthanide complex containing phen and nitrate ligands had a universal RE(NO_3_)_3_(phen)_2_ (RE = La, Ce, Pr, Nd, Lu, Gd, Tb) structure (Frechette *et al.*, 1992; Kepert *et al.*, 1996; Niu *et al.*, 1997; Wei *et al.*, 2002). However, the title [Tb(NO_3_)_4_(phen)]^-^ anion is the first example of ten-coordinated mononuclear lanthanide complex with one phen and four nitrate ligands. (Fig. 1)

There are several weak nontraditional C—H···O interactions which are significant in the crystal packing. The O(1), O(2), O(3), O(4), O(5), O(6), O(8), O(9), O(10) and O(11) atoms of the nitrates take part in constructing of C—H···O hydrogen bonds. The C(5)—H(5)···Cl(1) and C(26)—H(26)···O(4) hydrogen bonds link the [CuCl(phen))_2_]^+^ and [Tb(NO_3_)_4_(phen)]^-^ to form infinite cation and anion chains along the *a* direction, respectively.

Two cation chains join together to generate a double chain by obvious π–π stacking interaction between the 10-member rings of the [CuCl(phen))_2_]^+^ from the adjacent cation chains (*Cg*(1)···*Cg*(1)*^a^* = 3.635 (2) Å: *Cg*(1) [N(4)—C(22)—C(21)—C(20)—C(19)—C(18)—C(17)—C(16)—C(24)—C(23)]; symmetry code a: -*x* + 2, -*y* + 1, -*z*). Two anion chains attach each other also to generate a double chain similarly by C(33)—H(33)···O(1), C(29)—H(29)···O(11) hydrogen bonds and π–π stacking force between the adjacent anion chains (*Cg*(2)···*Cg*(2)*^b^* = 3.574 (2) Å: *Cg*(2) [C(28)—C(29)—C(30)—C(31)—C(35)— C(36)]; symmetry code b: -*x* + 1, -*y*, -*z* + 1). (Fig. 2)

Between the neighboring cation and anion chains, there are six weak intramolecular hydrogen bonds, *i.e.* C(2)—H(2)···O(2), C(14)—H(14)···O(3), C(6)—H(6)···O(5), C(9)—H(9)···O(6), C(22)—H(22)···O(9) and C(1)—H(1)···O(10), to assemble the cation and anion chains to a three-dimensional network. The 10-member rings of the phen in the [CuCl(phen))_2_]^+^ and [Tb(NO_3_)_4_(phen)]^-^ from the neighboring cation and anion chains are too close to result in π–π stacking effect (*Cg*(3)···*Cg*(4)*^b^* = 3.543 (2) Å: *Cg*(3) [N(2)—C(10)—C(9)— C(8)—C(7)—C(6)—C(5)—C(4)—C(12)—C(11)], *Cg*(4) [N(5)—C(25)—C(26)—C(27)—C(28)—C(29)—C(30)—C(31)—C(35)—C(36)]) (Fig. 3)

### Experimental

All the solvents and reagents were of analytical reagent grade and were used without further purification.2-(Carboxymethylthio) benzoic acid (2.0 mmol, 0.424 g)and phen (1.0 mmol, 0.198 g) were mixed in 30 ml me thanol and stirred for half an hour. Then Tb(NO_3_)_3_.6H_2_O (1.0 mmol, 0.453 g) and Cu(ClO)_2_.6H_2_O (1.0 mmol, 0.371 g) in 20 ml me thanol solution was added dropwise into the above solution and the mixture was carefully adjusted to pH = 5.5 by adding 0.1 mol/*L* NaOH methanol solution. The resulting mixture was left to react by stirring for 6 h at 80°C. The filtrate was slowly evaporated at ambient temperature for 3 days and block crystals were crystallized and isolated.

### Crystal data

**Table:**

[CuCl(C_12_H_8_N_2_)_2_][Tb(NO_3_)_4_(C_12_H_8_N_2_)]	*Z* = 2
*M**_r_* = 1046.56	*F*(000) = 1034
Triclinic, *P*1	*D*_x_ = 1.799 Mg m^−^^3^
*a* = 10.1556 (1) Å	Mo *K*α radiation, λ = 0.71073 Å
*b* = 13.4799 (2) Å	Cell parameters from 5578 reflections
*c* = 14.7710 (2) Å	θ = 2.7–23.4°
α = 81.103 (1)°	µ = 2.51 mm^−^^1^
β = 75.631 (1)°	*T* = 296 K
γ = 84.465 (1)°	Block, dark green
*V* = 1931.71 (4) Å^3^	0.21 × 0.20 × 0.12 mm

### Data collection

**Table:**

Brucker SMART APEX CCD diffractometer	8895 independent reflections
Radiation source: fine-focus sealed tube	6625 reflections with *I* > 2σ(*I*)
graphite	*R*_int_ = 0.040
φ and ω scans	θ_max_ = 27.9°, θ_min_ = 1.5°
Absorption correction: multi-scan (*SADABS*; Bruker, 2007)	*h* = −13→12
*T*_min_ = 0.620, *T*_max_ = 0.753	*k* = −17→17
25145 measured reflections	*l* = −19→19

### Refinement

**Table:**

Refinement on *F*^2^	Primary atom site location: structure-invariant direct methods
Least-squares matrix: full	Secondary atom site location: difference Fourier map
*R*[*F*^2^ > 2σ(*F*^2^)] = 0.035	Hydrogen site location: inferred from neighbouring sites
*wR*(*F*^2^) = 0.078	H-atom parameters constrained
*S* = 1.03	*w* = 1/[σ^2^(*F*_o_^2^) + (0.0324*P*)^2^ + 0.0379*P*] where *P* = (*F*_o_^2^ + 2*F*_c_^2^)/3
8895 reflections	(Δ/σ)_max_ = 0.002
550 parameters	Δρ_max_ = 0.44 e Å^−^^3^
0 restraints	Δρ_min_ = −0.75 e Å^−^^3^

### Special details

**Table:**

Geometry. All e.s.d.'s (except the e.s.d. in the dihedral angle between two l.s. planes) are estimated using the full covariance matrix. The cell e.s.d.'s are taken into account individually in the estimation of e.s.d.'s in distances, angles and torsion angles; correlations between e.s.d.'s in cell parameters are only used when they are defined by crystal symmetry. An approximate (isotropic) treatment of cell e.s.d.'s is used for estimating e.s.d.'s involving l.s. planes.
Refinement. Refinement of *F*^2^ against ALL reflections. The weighted *R*-factor *wR* and goodness of fit *S* are based on *F*^2^, conventional *R*-factors *R* are based on *F*, with *F* set to zero for negative *F*^2^. The threshold expression of *F*^2^ > σ(*F*^2^) is used only for calculating *R*-factors(gt) *etc*. and is not relevant to the choice of reflections for refinement. *R*-factors based on *F*^2^ are statistically about twice as large as those based on *F*, and *R*- factors based on ALL data will be even larger.

### Fractional atomic coordinates and isotropic or equivalent isotropic displacement parameters (Å^2^)

**Table:**

	*x*	*y*	*z*	*U*_iso_*/*U*_eq_	
Cu1	0.73762 (5)	0.32695 (3)	0.21343 (3)	0.05492 (13)	
Cl1	0.90518 (12)	0.23096 (9)	0.26516 (10)	0.0869 (4)	
Tb1	0.325455 (17)	0.785439 (13)	0.299634 (12)	0.04682 (7)	
O1	0.1163 (3)	0.78209 (19)	0.43529 (19)	0.0603 (7)	
O2	0.1843 (3)	0.64464 (19)	0.3777 (2)	0.0628 (7)	
O3	0.0066 (3)	0.6479 (2)	0.4951 (2)	0.0746 (8)	
O4	0.1129 (3)	0.8320 (2)	0.2462 (2)	0.0656 (7)	
O5	0.2875 (3)	0.9059 (2)	0.15931 (19)	0.0687 (8)	
O6	0.0900 (4)	0.9408 (4)	0.1263 (2)	0.1201 (15)	
O7	0.3373 (3)	0.6779 (2)	0.1801 (2)	0.0780 (9)	
O8	0.5124 (3)	0.7643 (3)	0.1568 (2)	0.0790 (9)	
O9	0.5033 (4)	0.6667 (4)	0.0564 (3)	0.1339 (17)	
O10	0.4917 (3)	0.6520 (2)	0.3407 (2)	0.0651 (7)	
O11	0.3846 (3)	0.7333 (2)	0.45410 (19)	0.0626 (7)	
O12	0.5408 (4)	0.6187 (3)	0.4772 (3)	0.1094 (13)	
N1	0.5261 (3)	0.3507 (2)	0.2544 (2)	0.0503 (7)	
N2	0.6844 (3)	0.2138 (2)	0.1598 (2)	0.0533 (8)	
N3	0.7869 (3)	0.4476 (2)	0.2587 (2)	0.0589 (8)	
N4	0.7720 (3)	0.4284 (2)	0.0837 (2)	0.0523 (8)	
N5	0.5271 (3)	0.8834 (2)	0.3029 (2)	0.0477 (7)	
N6	0.2649 (3)	0.9573 (2)	0.35658 (19)	0.0429 (6)	
N7	0.0995 (3)	0.6904 (2)	0.4384 (2)	0.0526 (8)	
N8	0.1612 (4)	0.8941 (3)	0.1752 (2)	0.0675 (9)	
N9	0.4544 (4)	0.7018 (3)	0.1283 (3)	0.0818 (11)	
N10	0.4742 (4)	0.6659 (3)	0.4257 (3)	0.0650 (9)	
C1	0.4478 (5)	0.4173 (3)	0.3046 (3)	0.0653 (11)	
H1	0.4885	0.4639	0.3275	0.078*	
C2	0.3047 (5)	0.4193 (3)	0.3240 (3)	0.0724 (12)	
H2	0.2522	0.4666	0.3596	0.087*	
C3	0.2437 (5)	0.3526 (4)	0.2912 (3)	0.0700 (12)	
H3	0.1491	0.3535	0.3050	0.084*	
C4	0.3211 (4)	0.2829 (3)	0.2372 (3)	0.0561 (10)	
C5	0.2666 (5)	0.2084 (3)	0.1998 (3)	0.0675 (12)	
H5	0.1727	0.2072	0.2094	0.081*	
C6	0.3466 (5)	0.1409 (4)	0.1519 (3)	0.0717 (13)	
H6	0.3073	0.0944	0.1278	0.086*	
C7	0.4906 (5)	0.1377 (3)	0.1363 (3)	0.0605 (11)	
C8	0.5823 (7)	0.0657 (4)	0.0913 (3)	0.0805 (14)	
H8	0.5495	0.0157	0.0674	0.097*	
C9	0.7168 (7)	0.0682 (4)	0.0822 (3)	0.0847 (15)	
H9	0.7767	0.0196	0.0531	0.102*	
C10	0.7661 (5)	0.1440 (3)	0.1167 (3)	0.0728 (12)	
H10	0.8596	0.1457	0.1092	0.087*	
C11	0.5482 (4)	0.2112 (3)	0.1704 (2)	0.0474 (8)	
C12	0.4641 (4)	0.2845 (3)	0.2209 (2)	0.0457 (8)	
C13	0.7994 (5)	0.4538 (4)	0.3447 (3)	0.0761 (13)	
H13	0.7719	0.4010	0.3924	0.091*	
C14	0.8516 (5)	0.5354 (4)	0.3664 (3)	0.0807 (14)	
H14	0.8595	0.5368	0.4276	0.097*	
C15	0.8917 (5)	0.6141 (4)	0.2983 (3)	0.0744 (13)	
H15	0.9274	0.6691	0.3127	0.089*	
C16	0.8789 (4)	0.6116 (3)	0.2065 (3)	0.0584 (10)	
C17	0.9179 (4)	0.6895 (3)	0.1299 (3)	0.0693 (12)	
H17	0.9526	0.7469	0.1404	0.083*	
C18	0.9057 (5)	0.6816 (3)	0.0426 (3)	0.0711 (12)	
H18	0.9298	0.7345	−0.0055	0.085*	
C19	0.8563 (4)	0.5937 (3)	0.0225 (3)	0.0559 (10)	
C20	0.8469 (5)	0.5776 (3)	−0.0671 (3)	0.0693 (12)	
H20	0.8751	0.6260	−0.1188	0.083*	
C21	0.7973 (5)	0.4923 (3)	−0.0791 (3)	0.0696 (12)	
H21	0.7876	0.4827	−0.1381	0.083*	
C22	0.7608 (4)	0.4188 (3)	−0.0009 (3)	0.0623 (11)	
H22	0.7268	0.3601	−0.0095	0.075*	
C23	0.8166 (4)	0.5157 (3)	0.0966 (3)	0.0490 (9)	
C24	0.8265 (4)	0.5257 (3)	0.1897 (3)	0.0519 (9)	
C25	0.6550 (4)	0.8495 (3)	0.2760 (3)	0.0591 (10)	
H25	0.6727	0.7914	0.2471	0.071*	
C26	0.7661 (4)	0.8961 (3)	0.2885 (3)	0.0624 (11)	
H26	0.8544	0.8686	0.2694	0.075*	
C27	0.7423 (4)	0.9814 (3)	0.3288 (3)	0.0561 (10)	
H27	0.8148	1.0140	0.3363	0.067*	
C28	0.6084 (4)	1.0210 (3)	0.3595 (2)	0.0445 (8)	
C29	0.5755 (4)	1.1092 (3)	0.4046 (2)	0.0504 (9)	
H29	0.6449	1.1429	0.4152	0.061*	
C30	0.4456 (4)	1.1444 (3)	0.4319 (2)	0.0511 (9)	
H30	0.4266	1.2020	0.4616	0.061*	
C31	0.3366 (4)	1.0959 (2)	0.4166 (2)	0.0432 (8)	
C32	0.1999 (4)	1.1325 (3)	0.4406 (2)	0.0504 (9)	
H32	0.1765	1.1897	0.4706	0.060*	
C33	0.1023 (4)	1.0843 (3)	0.4201 (3)	0.0522 (9)	
H33	0.0120	1.1094	0.4336	0.063*	
C34	0.1385 (4)	0.9968 (3)	0.3784 (2)	0.0495 (9)	
H34	0.0702	0.9642	0.3652	0.059*	
C35	0.3650 (3)	1.0073 (2)	0.3732 (2)	0.0383 (7)	
C36	0.5032 (3)	0.9685 (2)	0.3445 (2)	0.0398 (7)	

### Atomic displacement parameters (Å^2^)

**Table:**

	*U*^11^	*U*^22^	*U*^33^	*U*^12^	*U*^13^	*U*^23^
Cu1	0.0554 (3)	0.0529 (3)	0.0591 (3)	−0.0128 (2)	−0.0175 (2)	−0.0034 (2)
Cl1	0.0583 (7)	0.0772 (8)	0.1325 (11)	−0.0082 (6)	−0.0448 (7)	0.0025 (7)
Tb1	0.03933 (10)	0.04826 (11)	0.05639 (11)	−0.00717 (7)	−0.00697 (8)	−0.02226 (8)
O1	0.0607 (17)	0.0425 (16)	0.0709 (17)	−0.0083 (13)	0.0030 (14)	−0.0136 (12)
O2	0.0517 (16)	0.0542 (16)	0.0840 (19)	−0.0091 (13)	−0.0035 (15)	−0.0305 (14)
O3	0.070 (2)	0.072 (2)	0.0747 (19)	−0.0275 (16)	0.0032 (16)	−0.0080 (15)
O4	0.0483 (16)	0.082 (2)	0.0670 (18)	−0.0109 (14)	−0.0108 (14)	−0.0119 (15)
O5	0.0630 (19)	0.080 (2)	0.0635 (17)	−0.0186 (16)	−0.0085 (15)	−0.0123 (14)
O6	0.088 (3)	0.182 (4)	0.079 (2)	0.016 (3)	−0.029 (2)	0.018 (2)
O7	0.065 (2)	0.092 (2)	0.086 (2)	−0.0134 (17)	−0.0075 (18)	−0.0503 (17)
O8	0.0526 (17)	0.116 (3)	0.0743 (19)	−0.0143 (17)	−0.0015 (15)	−0.0485 (18)
O9	0.097 (3)	0.213 (5)	0.109 (3)	−0.002 (3)	−0.001 (2)	−0.117 (3)
O10	0.0594 (17)	0.0580 (17)	0.082 (2)	0.0019 (13)	−0.0165 (16)	−0.0275 (14)
O11	0.0644 (18)	0.0603 (17)	0.0678 (17)	−0.0034 (15)	−0.0151 (15)	−0.0243 (14)
O12	0.118 (3)	0.106 (3)	0.108 (3)	0.028 (2)	−0.053 (3)	−0.007 (2)
N1	0.0555 (19)	0.0413 (17)	0.0535 (18)	−0.0046 (15)	−0.0134 (15)	−0.0035 (14)
N2	0.054 (2)	0.0519 (19)	0.0513 (17)	−0.0056 (16)	−0.0080 (15)	−0.0062 (14)
N3	0.068 (2)	0.060 (2)	0.0545 (19)	−0.0167 (17)	−0.0223 (17)	−0.0029 (16)
N4	0.0579 (19)	0.0501 (18)	0.0532 (18)	−0.0121 (15)	−0.0207 (16)	−0.0029 (14)
N5	0.0354 (16)	0.0458 (17)	0.0629 (19)	−0.0023 (13)	−0.0077 (14)	−0.0168 (14)
N6	0.0401 (16)	0.0424 (16)	0.0480 (16)	−0.0043 (13)	−0.0104 (13)	−0.0103 (12)
N7	0.0415 (18)	0.056 (2)	0.061 (2)	−0.0089 (16)	−0.0091 (16)	−0.0105 (16)
N8	0.059 (2)	0.089 (3)	0.053 (2)	0.000 (2)	−0.0087 (19)	−0.0143 (19)
N9	0.063 (3)	0.115 (3)	0.079 (3)	0.003 (2)	−0.015 (2)	−0.058 (2)
N10	0.065 (2)	0.058 (2)	0.077 (3)	−0.0103 (19)	−0.022 (2)	−0.0108 (19)
C1	0.086 (3)	0.041 (2)	0.067 (3)	−0.002 (2)	−0.017 (2)	−0.0061 (19)
C2	0.075 (3)	0.060 (3)	0.068 (3)	0.020 (2)	−0.002 (2)	−0.006 (2)
C3	0.057 (3)	0.069 (3)	0.075 (3)	0.002 (2)	−0.013 (2)	0.012 (2)
C4	0.055 (2)	0.049 (2)	0.058 (2)	−0.0025 (19)	−0.015 (2)	0.0128 (18)
C5	0.062 (3)	0.064 (3)	0.080 (3)	−0.024 (2)	−0.032 (2)	0.016 (2)
C6	0.087 (4)	0.066 (3)	0.075 (3)	−0.030 (3)	−0.042 (3)	0.005 (2)
C7	0.084 (3)	0.055 (2)	0.047 (2)	−0.019 (2)	−0.021 (2)	0.0004 (18)
C8	0.121 (5)	0.069 (3)	0.058 (3)	−0.011 (3)	−0.024 (3)	−0.024 (2)
C9	0.115 (5)	0.069 (3)	0.064 (3)	0.004 (3)	−0.004 (3)	−0.024 (2)
C10	0.073 (3)	0.068 (3)	0.071 (3)	−0.005 (2)	0.001 (2)	−0.015 (2)
C11	0.055 (2)	0.045 (2)	0.0423 (19)	−0.0102 (17)	−0.0125 (17)	−0.0003 (15)
C12	0.052 (2)	0.039 (2)	0.0440 (19)	−0.0056 (16)	−0.0143 (17)	0.0069 (15)
C13	0.094 (3)	0.082 (3)	0.057 (2)	−0.023 (3)	−0.025 (2)	−0.003 (2)
C14	0.098 (4)	0.085 (3)	0.076 (3)	−0.014 (3)	−0.042 (3)	−0.020 (3)
C15	0.075 (3)	0.070 (3)	0.092 (3)	−0.010 (2)	−0.032 (3)	−0.030 (3)
C16	0.049 (2)	0.056 (2)	0.076 (3)	−0.0036 (19)	−0.017 (2)	−0.018 (2)
C17	0.067 (3)	0.046 (2)	0.095 (3)	−0.014 (2)	−0.014 (3)	−0.013 (2)
C18	0.070 (3)	0.049 (3)	0.088 (3)	−0.012 (2)	−0.012 (3)	0.003 (2)
C19	0.053 (2)	0.046 (2)	0.066 (2)	−0.0038 (18)	−0.014 (2)	0.0013 (18)
C20	0.078 (3)	0.062 (3)	0.063 (3)	−0.004 (2)	−0.023 (2)	0.015 (2)
C21	0.085 (3)	0.063 (3)	0.064 (3)	−0.006 (2)	−0.032 (2)	0.005 (2)
C22	0.070 (3)	0.061 (3)	0.063 (2)	−0.014 (2)	−0.028 (2)	−0.002 (2)
C23	0.042 (2)	0.047 (2)	0.056 (2)	−0.0047 (16)	−0.0126 (17)	−0.0020 (17)
C24	0.046 (2)	0.053 (2)	0.060 (2)	−0.0046 (17)	−0.0166 (18)	−0.0084 (18)
C25	0.043 (2)	0.050 (2)	0.087 (3)	0.0002 (18)	−0.011 (2)	−0.023 (2)
C26	0.035 (2)	0.059 (3)	0.093 (3)	−0.0011 (18)	−0.010 (2)	−0.017 (2)
C27	0.043 (2)	0.058 (3)	0.071 (3)	−0.0160 (18)	−0.022 (2)	0.001 (2)
C28	0.048 (2)	0.044 (2)	0.0452 (18)	−0.0127 (16)	−0.0180 (17)	0.0028 (15)
C29	0.066 (3)	0.042 (2)	0.050 (2)	−0.0185 (19)	−0.0233 (19)	−0.0013 (16)
C30	0.070 (3)	0.041 (2)	0.048 (2)	−0.0123 (19)	−0.0174 (19)	−0.0093 (15)
C31	0.056 (2)	0.0349 (18)	0.0374 (17)	−0.0040 (16)	−0.0097 (16)	−0.0031 (14)
C32	0.065 (3)	0.037 (2)	0.0433 (19)	0.0012 (18)	−0.0021 (18)	−0.0070 (15)
C33	0.049 (2)	0.045 (2)	0.054 (2)	0.0061 (17)	−0.0016 (18)	−0.0038 (17)
C34	0.037 (2)	0.054 (2)	0.056 (2)	−0.0020 (17)	−0.0083 (17)	−0.0099 (17)
C35	0.045 (2)	0.0351 (18)	0.0350 (16)	−0.0075 (15)	−0.0108 (15)	−0.0021 (13)
C36	0.0410 (19)	0.0366 (18)	0.0433 (18)	−0.0073 (15)	−0.0129 (15)	−0.0023 (14)

### Geometric parameters (Å, °)

**Table:**

Cu1—N2	1.997 (3)	C6—H6	0.9300
Cu1—N3	2.000 (3)	C7—C8	1.403 (6)
Cu1—N1	2.090 (3)	C7—C11	1.411 (5)
Cu1—N4	2.148 (3)	C8—C9	1.342 (7)
Cu1—Cl1	2.2513 (11)	C8—H8	0.9300
Tb1—O7	2.427 (3)	C9—C10	1.394 (7)
Tb1—O2	2.452 (3)	C9—H9	0.9300
Tb1—O10	2.457 (3)	C10—H10	0.9300
Tb1—O4	2.472 (3)	C11—C12	1.417 (5)
Tb1—O11	2.482 (3)	C13—C14	1.379 (6)
Tb1—O8	2.496 (3)	C13—H13	0.9300
Tb1—O5	2.513 (3)	C14—C15	1.360 (6)
Tb1—O1	2.531 (3)	C14—H14	0.9300
Tb1—N6	2.554 (3)	C15—C16	1.400 (6)
Tb1—N5	2.556 (3)	C15—H15	0.9300
O1—N7	1.257 (4)	C16—C24	1.399 (5)
O2—N7	1.269 (4)	C16—C17	1.425 (6)
O3—N7	1.217 (4)	C17—C18	1.346 (6)
O4—N8	1.262 (4)	C17—H17	0.9300
O5—N8	1.267 (4)	C18—C19	1.429 (6)
O6—N8	1.213 (4)	C18—H18	0.9300
O7—N9	1.284 (5)	C19—C20	1.400 (6)
O8—N9	1.251 (4)	C19—C23	1.406 (5)
O9—N9	1.202 (4)	C20—C21	1.352 (6)
O10—N10	1.264 (4)	C20—H20	0.9300
O11—N10	1.264 (4)	C21—C22	1.399 (5)
O12—N10	1.210 (4)	C21—H21	0.9300
N1—C1	1.328 (5)	C22—H22	0.9300
N1—C12	1.356 (4)	C23—C24	1.430 (5)
N2—C10	1.324 (5)	C25—C26	1.407 (5)
N2—C11	1.356 (5)	C25—H25	0.9300
N3—C13	1.324 (5)	C26—C27	1.350 (5)
N3—C24	1.363 (4)	C26—H26	0.9300
N4—C22	1.310 (5)	C27—C28	1.403 (5)
N4—C23	1.357 (4)	C27—H27	0.9300
N5—C25	1.319 (4)	C28—C36	1.415 (4)
N5—C36	1.357 (4)	C28—C29	1.424 (5)
N6—C34	1.323 (4)	C29—C30	1.343 (5)
N6—C35	1.362 (4)	C29—H29	0.9300
C1—C2	1.408 (6)	C30—C31	1.420 (5)
C1—H1	0.9300	C30—H30	0.9300
C2—C3	1.347 (6)	C31—C32	1.405 (5)
C2—H2	0.9300	C31—C35	1.413 (5)
C3—C4	1.384 (6)	C32—C33	1.353 (5)
C3—H3	0.9300	C32—H32	0.9300
C4—C12	1.414 (5)	C33—C34	1.391 (5)
C4—C5	1.433 (6)	C33—H33	0.9300
C5—C6	1.327 (6)	C34—H34	0.9300
C5—H5	0.9300	C35—C36	1.433 (5)
C6—C7	1.421 (6)		
			
N2—Cu1—N3	175.48 (12)	C8—C7—C11	116.3 (4)
N2—Cu1—N1	81.02 (13)	C8—C7—C6	125.6 (4)
N3—Cu1—N1	97.80 (13)	C11—C7—C6	118.1 (4)
N2—Cu1—N4	95.43 (12)	C9—C8—C7	120.8 (5)
N3—Cu1—N4	80.40 (12)	C9—C8—H8	119.6
N1—Cu1—N4	98.15 (11)	C7—C8—H8	119.6
N2—Cu1—Cl1	92.46 (9)	C8—C9—C10	119.6 (5)
N3—Cu1—Cl1	91.28 (10)	C8—C9—H9	120.2
N1—Cu1—Cl1	138.48 (8)	C10—C9—H9	120.2
N4—Cu1—Cl1	123.33 (9)	N2—C10—C9	122.2 (5)
O7—Tb1—O2	73.98 (10)	N2—C10—H10	118.9
O7—Tb1—O10	79.21 (10)	C9—C10—H10	118.9
O2—Tb1—O10	76.55 (9)	N2—C11—C7	122.5 (4)
O7—Tb1—O4	75.88 (10)	N2—C11—C12	116.7 (3)
O2—Tb1—O4	77.53 (9)	C7—C11—C12	120.7 (4)
O10—Tb1—O4	148.03 (9)	N1—C12—C4	123.1 (4)
O7—Tb1—O11	126.04 (10)	N1—C12—C11	117.6 (3)
O2—Tb1—O11	74.19 (9)	C4—C12—C11	119.2 (4)
O10—Tb1—O11	51.60 (9)	N3—C13—C14	122.5 (4)
O4—Tb1—O11	135.66 (10)	N3—C13—H13	118.7
O7—Tb1—O8	51.71 (10)	C14—C13—H13	118.7
O2—Tb1—O8	120.01 (10)	C15—C14—C13	120.1 (4)
O10—Tb1—O8	70.74 (11)	C15—C14—H14	120.0
O4—Tb1—O8	107.45 (10)	C13—C14—H14	120.0
O11—Tb1—O8	116.13 (10)	C14—C15—C16	119.5 (4)
O7—Tb1—O5	76.13 (10)	C14—C15—H15	120.2
O2—Tb1—O5	125.16 (9)	C16—C15—H15	120.2
O10—Tb1—O5	139.48 (10)	C24—C16—C15	117.1 (4)
O4—Tb1—O5	50.96 (9)	C24—C16—C17	118.6 (4)
O11—Tb1—O5	156.26 (9)	C15—C16—C17	124.2 (4)
O8—Tb1—O5	68.75 (10)	C18—C17—C16	121.4 (4)
O7—Tb1—O1	117.94 (10)	C18—C17—H17	119.3
O2—Tb1—O1	50.84 (9)	C16—C17—H17	119.3
O10—Tb1—O1	108.47 (9)	C17—C18—C19	121.4 (4)
O4—Tb1—O1	67.45 (9)	C17—C18—H18	119.3
O11—Tb1—O1	68.22 (9)	C19—C18—H18	119.3
O8—Tb1—O1	169.63 (9)	C20—C19—C23	116.5 (4)
O5—Tb1—O1	111.39 (9)	C20—C19—C18	124.9 (4)
O7—Tb1—N6	149.35 (10)	C23—C19—C18	118.6 (4)
O2—Tb1—N6	119.26 (9)	C21—C20—C19	120.7 (4)
O10—Tb1—N6	129.21 (9)	C21—C20—H20	119.7
O4—Tb1—N6	80.28 (9)	C19—C20—H20	119.7
O11—Tb1—N6	84.57 (9)	C20—C21—C22	118.6 (4)
O8—Tb1—N6	120.49 (10)	C20—C21—H21	120.7
O5—Tb1—N6	73.93 (9)	C22—C21—H21	120.7
O1—Tb1—N6	68.42 (8)	N4—C22—C21	123.3 (4)
O7—Tb1—N5	122.08 (10)	N4—C22—H22	118.3
O2—Tb1—N5	145.30 (9)	C21—C22—H22	118.3
O10—Tb1—N5	77.03 (9)	N4—C23—C19	122.7 (3)
O4—Tb1—N5	133.72 (9)	N4—C23—C24	117.6 (3)
O11—Tb1—N5	71.84 (9)	C19—C23—C24	119.7 (3)
O8—Tb1—N5	70.66 (10)	N3—C24—C16	122.6 (3)
O5—Tb1—N5	89.50 (9)	N3—C24—C23	117.1 (3)
O1—Tb1—N5	119.54 (9)	C16—C24—C23	120.3 (3)
N6—Tb1—N5	64.30 (9)	N5—C25—C26	123.7 (4)
C1—N1—C12	118.0 (3)	N5—C25—H25	118.2
C13—N3—C24	118.1 (3)	C26—C25—H25	118.2
C22—N4—C23	118.2 (3)	C27—C26—C25	118.9 (4)
C25—N5—C36	117.4 (3)	C27—C26—H26	120.6
C34—N6—C35	117.8 (3)	C25—C26—H26	120.6
O3—N7—O1	122.4 (3)	C26—C27—C28	120.1 (3)
O3—N7—O2	121.8 (3)	C26—C27—H27	120.0
O1—N7—O2	115.8 (3)	C28—C27—H27	120.0
O6—N8—O4	121.8 (4)	C27—C28—C36	117.0 (3)
O6—N8—O5	122.2 (4)	C27—C28—C29	123.1 (3)
O4—N8—O5	116.0 (3)	C36—C28—C29	119.9 (3)
O9—N9—O8	122.7 (5)	C30—C29—C28	120.8 (3)
O9—N9—O7	121.5 (4)	C30—C29—H29	119.6
O8—N9—O7	115.8 (4)	C28—C29—H29	119.6
O12—N10—O11	121.3 (4)	C29—C30—C31	121.5 (3)
O12—N10—O10	122.2 (4)	C29—C30—H30	119.2
O11—N10—O10	116.5 (3)	C31—C30—H30	119.2
N1—C1—C2	121.7 (4)	C32—C31—C35	117.4 (3)
N1—C1—H1	119.1	C32—C31—C30	123.3 (3)
C2—C1—H1	119.1	C35—C31—C30	119.3 (3)
C3—C2—C1	120.1 (4)	C33—C32—C31	119.8 (3)
C3—C2—H2	120.0	C33—C32—H32	120.1
C1—C2—H2	120.0	C31—C32—H32	120.1
C2—C3—C4	120.3 (4)	C32—C33—C34	119.3 (3)
C2—C3—H3	119.9	C32—C33—H33	120.4
C4—C3—H3	119.9	C34—C33—H33	120.4
C3—C4—C12	116.8 (4)	N6—C34—C33	123.5 (3)
C3—C4—C5	124.7 (4)	N6—C34—H34	118.2
C12—C4—C5	118.4 (4)	C33—C34—H34	118.2
C6—C5—C4	121.7 (4)	N6—C35—C31	122.1 (3)
C6—C5—H5	119.1	N6—C35—C36	118.3 (3)
C4—C5—H5	119.1	C31—C35—C36	119.6 (3)
C5—C6—C7	121.8 (4)	N5—C36—C28	123.0 (3)
C5—C6—H6	119.1	N5—C36—C35	118.2 (3)
C7—C6—H6	119.1	C28—C36—C35	118.8 (3)
			
O7—Tb1—O1—N7	34.0 (2)	O5—Tb1—N5—C36	81.5 (2)
O2—Tb1—O1—N7	0.59 (18)	O1—Tb1—N5—C36	−33.0 (3)
O10—Tb1—O1—N7	−53.4 (2)	N6—Tb1—N5—C36	9.1 (2)
O4—Tb1—O1—N7	92.7 (2)	O7—Tb1—N6—C34	66.3 (3)
O11—Tb1—O1—N7	−86.4 (2)	O2—Tb1—N6—C34	−42.6 (3)
O8—Tb1—O1—N7	30.5 (6)	O10—Tb1—N6—C34	−139.1 (2)
O5—Tb1—O1—N7	119.18 (19)	O4—Tb1—N6—C34	27.1 (2)
N6—Tb1—O1—N7	−179.2 (2)	O11—Tb1—N6—C34	−111.1 (3)
N5—Tb1—O1—N7	−138.63 (19)	O8—Tb1—N6—C34	131.7 (2)
O7—Tb1—O2—N7	−150.2 (2)	O5—Tb1—N6—C34	79.1 (3)
O10—Tb1—O2—N7	127.4 (2)	O1—Tb1—N6—C34	−42.4 (2)
O4—Tb1—O2—N7	−71.52 (19)	N5—Tb1—N6—C34	176.5 (3)
O11—Tb1—O2—N7	73.9 (2)	O7—Tb1—N6—C35	−119.5 (2)
O8—Tb1—O2—N7	−174.64 (18)	O2—Tb1—N6—C35	131.6 (2)
O5—Tb1—O2—N7	−90.7 (2)	O10—Tb1—N6—C35	35.1 (3)
O1—Tb1—O2—N7	−0.58 (17)	O4—Tb1—N6—C35	−158.7 (2)
N6—Tb1—O2—N7	−0.3 (2)	O11—Tb1—N6—C35	63.1 (2)
N5—Tb1—O2—N7	86.0 (2)	O8—Tb1—N6—C35	−54.1 (2)
O7—Tb1—O4—N8	−84.2 (2)	O5—Tb1—N6—C35	−106.7 (2)
O2—Tb1—O4—N8	−160.5 (2)	O1—Tb1—N6—C35	131.8 (2)
O10—Tb1—O4—N8	−124.1 (3)	N5—Tb1—N6—C35	−9.4 (2)
O11—Tb1—O4—N8	148.2 (2)	C12—N1—C1—C2	0.8 (5)
O8—Tb1—O4—N8	−42.7 (3)	N1—C1—C2—C3	−0.2 (6)
O5—Tb1—O4—N8	−0.7 (2)	C1—C2—C3—C4	−0.9 (6)
O1—Tb1—O4—N8	146.9 (2)	C2—C3—C4—C12	1.4 (5)
N6—Tb1—O4—N8	76.4 (2)	C2—C3—C4—C5	179.3 (4)
N5—Tb1—O4—N8	37.0 (3)	C3—C4—C5—C6	−177.1 (4)
O7—Tb1—O5—N8	83.7 (2)	C12—C4—C5—C6	0.8 (5)
O2—Tb1—O5—N8	25.1 (3)	C4—C5—C6—C7	1.1 (6)
O10—Tb1—O5—N8	137.8 (2)	C5—C6—C7—C8	176.2 (4)
O4—Tb1—O5—N8	0.7 (2)	C5—C6—C7—C11	−2.3 (6)
O11—Tb1—O5—N8	−115.6 (3)	C11—C7—C8—C9	0.1 (6)
O8—Tb1—O5—N8	137.6 (3)	C6—C7—C8—C9	−178.4 (4)
O1—Tb1—O5—N8	−31.3 (3)	C7—C8—C9—C10	−1.0 (7)
N6—Tb1—O5—N8	−89.7 (2)	C11—N2—C10—C9	−0.1 (6)
N5—Tb1—O5—N8	−153.0 (2)	C8—C9—C10—N2	1.0 (7)
O2—Tb1—O7—N9	−153.2 (3)	C10—N2—C11—C7	−0.7 (5)
O10—Tb1—O7—N9	−74.3 (3)	C10—N2—C11—C12	177.1 (3)
O4—Tb1—O7—N9	126.0 (3)	C8—C7—C11—N2	0.7 (5)
O11—Tb1—O7—N9	−97.2 (3)	C6—C7—C11—N2	179.4 (3)
O8—Tb1—O7—N9	−0.4 (2)	C8—C7—C11—C12	−177.0 (3)
O5—Tb1—O7—N9	73.3 (3)	C6—C7—C11—C12	1.6 (5)
O1—Tb1—O7—N9	−179.6 (2)	C1—N1—C12—C4	−0.3 (5)
N6—Tb1—O7—N9	85.9 (3)	C1—N1—C12—C11	−177.7 (3)
N5—Tb1—O7—N9	−7.1 (3)	C3—C4—C12—N1	−0.8 (5)
O7—Tb1—O8—N9	0.4 (2)	C5—C4—C12—N1	−178.9 (3)
O2—Tb1—O8—N9	30.8 (3)	C3—C4—C12—C11	176.6 (3)
O10—Tb1—O8—N9	91.7 (3)	C5—C4—C12—C11	−1.5 (5)
O4—Tb1—O8—N9	−54.6 (3)	N2—C11—C12—N1	−0.1 (4)
O11—Tb1—O8—N9	117.0 (3)	C7—C11—C12—N1	177.8 (3)
O5—Tb1—O8—N9	−88.4 (3)	N2—C11—C12—C4	−177.6 (3)
O1—Tb1—O8—N9	4.3 (7)	C7—C11—C12—C4	0.3 (5)
N6—Tb1—O8—N9	−143.4 (2)	C24—N3—C13—C14	−0.5 (7)
N5—Tb1—O8—N9	174.3 (3)	N3—C13—C14—C15	0.5 (8)
O7—Tb1—O10—N10	−157.5 (2)	C13—C14—C15—C16	0.3 (7)
O2—Tb1—O10—N10	−81.6 (2)	C14—C15—C16—C24	−1.1 (6)
O4—Tb1—O10—N10	−118.2 (2)	C14—C15—C16—C17	−179.8 (4)
O11—Tb1—O10—N10	−1.2 (2)	C24—C16—C17—C18	−0.2 (6)
O8—Tb1—O10—N10	149.5 (2)	C15—C16—C17—C18	178.6 (4)
O5—Tb1—O10—N10	149.3 (2)	C16—C17—C18—C19	−1.7 (7)
O1—Tb1—O10—N10	−41.4 (2)	C17—C18—C19—C20	−176.6 (4)
N6—Tb1—O10—N10	35.4 (3)	C17—C18—C19—C23	1.8 (6)
N5—Tb1—O10—N10	75.7 (2)	C23—C19—C20—C21	2.3 (6)
O7—Tb1—O11—N10	30.5 (3)	C18—C19—C20—C21	−179.2 (4)
O2—Tb1—O11—N10	86.4 (2)	C19—C20—C21—C22	−2.6 (7)
O10—Tb1—O11—N10	1.24 (19)	C23—N4—C22—C21	2.5 (6)
O4—Tb1—O11—N10	138.8 (2)	C20—C21—C22—N4	0.1 (7)
O8—Tb1—O11—N10	−29.7 (2)	C22—N4—C23—C19	−2.7 (6)
O5—Tb1—O11—N10	−126.2 (3)	C22—N4—C23—C24	179.1 (3)
O1—Tb1—O11—N10	140.0 (2)	C20—C19—C23—N4	0.3 (6)
N6—Tb1—O11—N10	−151.1 (2)	C18—C19—C23—N4	−178.2 (4)
N5—Tb1—O11—N10	−86.4 (2)	C20—C19—C23—C24	178.5 (4)
N2—Cu1—N1—C1	177.3 (3)	C18—C19—C23—C24	0.0 (6)
N3—Cu1—N1—C1	−7.1 (3)	C13—N3—C24—C16	−0.3 (6)
N4—Cu1—N1—C1	−88.5 (3)	C13—N3—C24—C23	177.8 (4)
Cl1—Cu1—N1—C1	93.8 (3)	C15—C16—C24—N3	1.1 (6)
N2—Cu1—N1—C12	−3.3 (2)	C17—C16—C24—N3	179.9 (4)
N3—Cu1—N1—C12	172.3 (2)	C15—C16—C24—C23	−177.0 (4)
N4—Cu1—N1—C12	90.9 (2)	C17—C16—C24—C23	1.9 (6)
Cl1—Cu1—N1—C12	−86.8 (2)	N4—C23—C24—N3	−1.6 (5)
N1—Cu1—N2—C10	−176.6 (3)	C19—C23—C24—N3	−179.9 (3)
N4—Cu1—N2—C10	86.0 (3)	N4—C23—C24—C16	176.5 (3)
Cl1—Cu1—N2—C10	−37.9 (3)	C19—C23—C24—C16	−1.7 (5)
N1—Cu1—N2—C11	3.4 (2)	C36—N5—C25—C26	0.4 (6)
N4—Cu1—N2—C11	−94.1 (2)	N5—C25—C26—C27	−1.2 (6)
Cl1—Cu1—N2—C11	142.1 (2)	C25—C26—C27—C28	1.5 (6)
N1—Cu1—N3—C13	86.2 (4)	C26—C27—C28—C36	−1.1 (5)
N4—Cu1—N3—C13	−176.8 (4)	C26—C27—C28—C29	178.5 (3)
Cl1—Cu1—N3—C13	−53.2 (4)	C27—C28—C29—C30	179.4 (3)
N1—Cu1—N3—C24	−102.1 (3)	C36—C28—C29—C30	−1.0 (5)
N4—Cu1—N3—C24	−5.1 (3)	C28—C29—C30—C31	−0.4 (5)
Cl1—Cu1—N3—C24	118.5 (3)	C29—C30—C31—C32	−177.3 (3)
N2—Cu1—N4—C22	0.4 (4)	C29—C30—C31—C35	1.2 (5)
N3—Cu1—N4—C22	−177.9 (4)	C35—C31—C32—C33	−1.4 (5)
N1—Cu1—N4—C22	−81.3 (4)	C30—C31—C32—C33	177.2 (3)
Cl1—Cu1—N4—C22	96.9 (4)	C31—C32—C33—C34	2.4 (5)
N2—Cu1—N4—C23	−177.7 (3)	C35—N6—C34—C33	−2.2 (5)
N3—Cu1—N4—C23	4.1 (2)	C32—C33—C34—N6	−0.5 (5)
N1—Cu1—N4—C23	100.7 (2)	C34—N6—C35—C31	3.2 (4)
Cl1—Cu1—N4—C23	−81.1 (3)	C34—N6—C35—C36	−176.3 (3)
O7—Tb1—N5—C25	−33.4 (3)	C32—C31—C35—N6	−1.4 (4)
O2—Tb1—N5—C25	76.0 (3)	C30—C31—C35—N6	180.0 (3)
O10—Tb1—N5—C25	34.8 (3)	C32—C31—C35—C36	178.0 (3)
O4—Tb1—N5—C25	−135.0 (3)	C30—C31—C35—C36	−0.6 (4)
O11—Tb1—N5—C25	88.3 (3)	C25—N5—C36—C28	0.1 (5)
O8—Tb1—N5—C25	−39.1 (3)	C25—N5—C36—C35	179.2 (3)
O5—Tb1—N5—C25	−106.7 (3)	C27—C28—C36—N5	0.3 (5)
O1—Tb1—N5—C25	138.9 (3)	C29—C28—C36—N5	−179.3 (3)
N6—Tb1—N5—C25	−179.0 (3)	C27—C28—C36—C35	−178.8 (3)
O7—Tb1—N5—C36	154.7 (2)	C29—C28—C36—C35	1.6 (4)
O2—Tb1—N5—C36	−95.8 (3)	N6—C35—C36—N5	−0.5 (4)
O10—Tb1—N5—C36	−137.0 (2)	C31—C35—C36—N5	−179.9 (3)
O4—Tb1—N5—C36	53.1 (3)	N6—C35—C36—C28	178.7 (3)
O11—Tb1—N5—C36	−83.6 (2)	C31—C35—C36—C28	−0.8 (4)
O8—Tb1—N5—C36	149.1 (3)		

### Hydrogen-bond geometry (Å, °)

**Table:**

*D*—H···*A*	*D*—H	H···*A*	*D*···*A*	*D*—H···*A*
C5—H5···Cl1^i^	0.93	2.64	3.551 (5)	167
C33—H33···O1^ii^	0.93	2.62	3.287 (5)	130
C2—H2···O2	0.93	2.47	3.302 (5)	149
C14—H14···O3^iii^	0.93	2.67	3.350 (5)	131
C26—H26···O4^iii^	0.93	2.57	3.468 (5)	162
C6—H6···O5^iv^	0.93	2.53	3.261 (5)	136
C9—H9···O6^v^	0.93	2.66	3.228 (6)	120
C22—H22···O9^v^	0.93	2.67	3.342 (6)	130
C1—H1···O10	0.93	2.58	3.377 (5)	144
C29—H29···O11^vi^	0.93	2.69	3.314 (4)	126

Symmetry codes: (i) *x*−1, *y*, *z*; (ii) −*x*, −*y*+2, −*z*+1; (iii) *x*+1, *y*, *z*; (iv) *x*, *y*−1, *z*; (v) −*x*+1, −*y*+1, −*z*; (vi) −*x*+1, −*y*+2, −*z*+1.
